# Supplementary material for: Pleiotropic Effects of Functional MUC1 Variants on Cardiometabolic, Renal, and Hematological Traits in the Taiwanese Population
Source: Int J Mol Sci. 2021 Sep 30;22(19):10641. doi: 10.3390/ijms221910641 (PMC8509060; doi:10.3390/ijms221910641)

Supplementary Method

Definitions of hypertension, diabetes mellitus, obesity, and current smoking

Hypertension was defined as a systolic blood pressure (BP) of ≥140 mmHg and/or a diastolic BP of ≥90 mmHg or a self-reported history of hypertension. Diabetes mellitus (DM) was defined as a fasting plasma glucose level of ≥126 mg/dL, a glycohemoglobin (HbA1C) value of ≥6.5%, or a self-reported history of DM. Hyperlipidemia was defined as a total cholesterol level of >200 mg/dL, a triglyceride level of >200 mg/dL, or a self-reported history of hyperlipidemia. Obesity was defined if the body mass index (BMI) was ≥25 kg/m^2. Current smoking was defined as smoking cigarettes regularly at the time of survey.

Table S1. Association between *MUC1* rs12411216 and rs4072037 genotypes and atherosclerotic risk factors

rs12411216 genotypes	CC	CA	AA	beta	SE	*P* value*	*Adjusted P* value
Diabetes mellitus	9.58% (4724)	9.44% (2589)	9.09% (362)	-0.0292	0.0216	0.1771	0.999
Hypertension	21.99% (10843)	23.14% (6349)	22.03% (877)	0.0394	0.0159	0.0132	0.132
Current smoking	9.05% (4463)	9.15% (2510)	9.22% (367)	0.0048	0.0219	0.8259	0.999
Gout	3.68% (1813)	4.18% (1146)	5.38% (214)	0.1714	0.0309	2.94×10^{-8}	2.94×10^{-7}
Microalbuminuria	11.52% (5678)	11.12% (3051)	10.35% (412)	-0.0531	0.0194	0.0062	0.062
**rs4072037 genotypes**	**AA**	**GA**	**GG**	**beta**	**SE**	***P* value***	***Adjusted P* value**
Diabetes mellitus	9.58% (4691)	9.43% (2611)	9.16% (373)	-0.0273	0.0215	0.204	0.999
Hypertension	21.96% (10752)	23.17% (6416)	22.12% (901)	0.044	0.0158	0.0054	0.054
Current smoking	9.05% (4433)	9.15% (2533)	9.18% (374)	0.0048	0.0218	0.8244	0.999
Gout	3.68% (1801)	4.17% (1155)	5.33% (217)	0.1697	0.0308	3.66×10^{-8}	3.66×10^{-7}
Microalbuminuria	11.53% (5645)	11.10% (3075)	10.34% (421)	-0.0552	0.0193	0.0043	0.043

* *P* value adjusted for age, sex, body mass index and current smoking
Current smoking: adjusted for age, BMI and sex
Adjusted *P* value: with Bonferroni correction, n = 10

Table S2. Association between *MUC1* rs12411216 and rs4072037 genotypes and metabolic and hematological phenotypes according to sex

		rs12411216 genotypes				rs4072037 genotypes			
		beta	SE	*P1* value	*t test*	beta	SE	*P1* value	*t test*
Waist circumference (cm)	Male	-0.1479	0.0436	0.0007	-1.0960	-0.1513	0.0434	0.0005	-1.1713
	Female	-0.0825	0.0408	0.0429		-0.0817	0.0406	0.0442	
Creatinine (mg/dL)	Male	0.0064	0.0028	0.0216	0.2330	0.0066	0.0028	0.0189	0.2214
	Female	0.0057	0.0012	3.00×10^{-6}		0.0059	0.0012	1.00×10^{-6}	
eGFR (mL/min/1.73 m^2)	Male	-0.8235	0.1832	7.00×10^{-6}	1.6997	-0.8437	0.1826	4.00×10^{-6}	1.7917
	Female	-1.2528	0.1738	5.76×10^{-13}		-1.2944	0.1730	7.49×10^{-14}	
BUN (mg/L)	Male	0.2366	0.0378	3.89×10^{-10}	-0.6822	0.2337	0.0377	5.48×10^{-10}	-0.8546
	Female	0.2675	0.0250	1.00×10^{-26}		0.2722	0.0249	7.29×10^{-28}	
Hematocrit (%)	Male	-0.1254	0.0340	0.0002	0.6857	-0.1211	0.0338	0.0003	0.7824
	Female	-0.1547	0.0260	2.75×10^{-9}		-0.1544	0.0259	2.51×10^{-9}	
Red blood cell count (10^6/μL)	Male	-0.0112	0.0046	0.0157	0.9549	-0.0107	0.0046	0.0207	0.9338
	Female	-0.0166	0.0032	1.58×10^{-7}		-0.0159	0.0031	4.19×10^{-7}	
Albuminuria (mg/L)	Male	-0.0259	0.0047	3.68×10^{-8}	-2.6977*	-0.0260	0.0047	2.87×10^{-8}	-2.7698*
	Female	-0.0104	0.0033	0.0019		-0.0101	0.0033	0.0023	
Hemoglobin (g/dL)	Male	-0.0542	0.0115	2.00×10^{-6}	-0.3233	-0.0518	0.0114	6.00×10^{-6}	-0.1541
	Female	-0.0494	0.0094	1.33×10^{-7}		-0.0496	0.0093	1.08×10^{-7}	
Uric acid (mg/dL)	Male	0.0770	0.0127	1.26×10^{-9}	0.7006	0.0800	0.0126	2.34×10^{-10}	0.8579
	Female	0.0666	0.0076	2.73×10^{-18}		0.0674	0.0076	7.74×10^{-19}	

* $P < 0.01$
Abbreviations as in Table 1.
P1 value adjusted for age, body mass index and current smoking

Table S3. Association between *MUC1* rs12411216 and rs4072037 genotypes and atherosclerotic risk factors: according to sex

		rs12411216 genotypes				rs4072037 genotypes			
	Sex	beta	SE	*P1* value	*t test*	beta	SE	*P1* value	*t test*
Diabetes mellitus (%)	Male	-0.0302	0.0318	0.3430	-0.0305	-0.0292	0.0317	0.3575	-0.0650
	Female	-0.0289	0.0295	0.3270		-0.0264	0.0293	0.3681	
Current smoking (%)	Male	0.0090	0.0248	0.7161	0.4958	0.0103	0.0247	0.6769	0.6102
	Female	-0.0170	0.0463	0.7132		-0.0216	0.0461	0.6391	
Gout (%)	Male	0.1600	0.0327	1.00×10^{-6}	-1.0768	0.1592	0.0326	1.00×10^{-6}	-1.0109
	Female	0.2659	0.0927	0.0041		0.2584	0.0925	0.0052	
Microalbuminuria (%)	Male	-0.0921	0.0317	0.0036	-1.5182	-0.0939	0.0316	0.0029	-1.5017
	Female	-0.0312	0.0246	0.2048		-0.0338	0.0245	0.1680	

P1 value adjusted for age, body mass index and current smoking

Table S4. Summary of coefficients used for Mendelian randomization analysis: *MUC1* rs12411216 genotypes (G_A) and related phenotypes

T_A	T_B	T_A-T_B			G_A-T_A			G_A-T_B			IV_A-T_B (IV_A-T_B-AdjT_A)		
		BETA	SE	*P**	BETA	SE	*P**	BETA	SE	*P**	BETA	SE	*P**(*P***)
eGFR	Hematocrit	-0.0112	0.0006	2.88×10^{-87}	-1.0751	0.1294	9.86×10^{-17}	-0.1509	0.0209	5.07×10^{-13}	0.1404	0.0194	5.07×10^{-13} (5.12×10^{-15})
	Uric acid	-0.0150	0.0002	$< 10^{-307}$	-1.0145	0.1323	1.73×10^{-14}	0.0690	0.0067	5.73×10^{-25}	-0.0681	0.0066	5.73×10^{-25} (3.68×10^{-17})
	Gout	-0.0248	0.0011	4.90×10^{-118}	-1.0751	0.1294	9.86×10^{-17}	0.1714	0.0309	2.94×10^{-8}	-0.1594	0.0287	2.94×10^{-8} (1.00×10^{-6})
	Albuminuria	-0.0015	0.0001	3.37×10^{-90}	-1.0751	0.1294	9.86×10^{-17}	-0.0159	0.0027	5.31×10^{-9}	0.0148	0.0025	5.31×10^{-9} (1.16×10^{-10})
Hematocrit	eGFR	-0.4312	0.0217	2.88×10^{-87}	-0.1509	0.0209	5.07×10^{-13}	-1.0751	0.1294	9.86×10^{-17}	7.1198	0.8570	9.86×10^{-17} (1.01×10^{-18})
	Uric acid	0.0405	0.0011	1.19×10^{-285}	-0.1503	0.0213	1.76×10^{-12}	0.0690	0.0067	5.73×10^{-25}	-0.4603	0.0446	5.73×10^{-25} (9.32×10^{-30})
	Gout	-0.0108	0.0054	0.0467	-0.1509	0.0209	5.07×10^{-13}	0.1714	0.0309	2.94×10^{-8}	-1.1348	0.2046	2.94×10^{-8} (3.68×10^{-8})
	Albuminuria	0.0003	0.0005	0.5090	-0.1509	0.0209	5.07×10^{-13}	-0.0159	0.0027	5.31×10^{-9}	0.1056	0.0181	5.31×10^{-9} (5.81×10^{-9})
Uric acid	eGFR	-0.0150	0.0002	$< 10^{-307}$	0.0690	0.0067	5.73×10^{-25}	-1.0145	0.1323	1.73×10^{-14}	-14.7023	1.9167	1.73×10^{-14} (1.00×10^{-16})
	Hematocrit	0.0405	0.0011	1.19×10^{-285}	0.0690	0.0067	5.73×10^{-25}	-0.1503	0.0213	1.76×10^{-12}	-2.1790	0.3089	1.76×10^{-12} (2.63×10^{-17})
	Gout	0.6509	0.0146	$< 10^{-307}$	0.0767	0.0068	3.94×10^{-25}	0.1714	0.0309	2.94×10^{-8}	2.2253	0.4013	2.94×10^{-8} (0.0004)
	Albuminuria	0.1308	0.0087	1.07×10^{-50}	0.0690	0.0067	5.73×10^{-25}	-0.0157	0.0027	1.09×10^{-8}	-0.2278	0.0398	1.09×10^{-8} (3.30×10^{-10})
Gout	eGFR	-7.9984	0.4028	1.53×10^{-87}	0.1714	0.0309	2.94×10^{-8}	-1.0751	0.1294	9.86×10^{-17}	-6.2871	0.7567	9.86×10^{-17} (1.91×10^{-15})
	Hematocrit	-0.2942	0.0652	6.00×10^{-6}	0.1714	0.0309	2.94×10^{-8}	-0.1509	0.0209	5.07×10^{-13}	-0.8827	0.1222	5.07×10^{-13} (9.40×10^{-13})
	Uric acid	1.0984	0.0210	$< 10^{-307}$	0.1714	0.0309	2.94×10^{-8}	0.0767	0.0068	3.94×10^{-29}	0.4486	0.0400	3.94×10^{-29} (2.75×10^{-25})
	Albuminuria	0.0725	0.0085	1.67×10^{-17}	0.1714	0.0309	2.94×10^{-8}	-0.0159	0.0027	5.31×10^{-9}	-0.0932	0.0160	5.31×10^{-9} (1.93×10^{-9})
Albuminuria	eGFR	-3.3546	0.1664	3.37×10^{-90}	-0.0159	0.0027	5.31×10^{-9}	-1.0751	0.1294	9.86×10^{-17}	67.1935	8.0876	9.86×10^{-17} (2.26×10^{-18})
	Hematocrit	0.0178	0.0269	0.5090	-0.0159	0.0027	5.31×10^{-9}	-0.1509	0.0209	5.07×10^{-13}	9.4339	1.3058	5.07×10^{-13} (5.54×10^{-13})
	Uric acid	0.1308	0.0087	1.07×10^{-50}	-0.0157	0.0027	1.09×10^{-8}	0.0690	0.0067	5.73×10^{-25}	-4.3150	0.4180	5.73×10^{-25} (3.48×10^{-26})
	Gout	0.1856	0.0361	2.63×10^{-7}	-0.0159	0.0027	5.31×10^{-9}	0.1714	0.0309	2.94×10^{-8}	-10.7094	1.9314	2.94×10^{-8} (1.21×10^{-8})

Abbreviations as in Table 1.

IV_A: Instrumental variables for G_A.

*Adjusted for age, sex, smoking, and body mass index (BMI).

**After further adjustment of T_A

Table S5. Summary of coefficients used for Mendelian randomization analysis: *MUC1* rs4072037 genotypes (G_A) and related phenotypes

T_A	T_B	T_A-T_B			G_A-T_A			G_A-T_B			IV_A-T_B (IV_A-T_B-AdjT_A)		
		BETA	SE	*P**	BETA	SE	*P**	BETA	SE	*P**	BETA	SE	*P**(*P***)
eGFR	Hematocrit	-0.0112	0.0006	2.88×10^{-87}	-1.1074	0.1289	8.51×10^{-18}	-0.1497	0.0208	6.34×10^{-13}	0.1352	0.01879	6.34×10^{-13} (5.55×10^{-15})
	Uric acid	-0.0150	0.0002	$< 10^{-307}$	-1.0485	0.1317	1.71×10^{-15}	0.0704	0.0067	4.02×10^{-26}	-0.0671	0.00635	4.02×10^{-26} (8.25×10^{-18})
	Gout	-0.0248	0.0011	4.90×10^{-118}	-1.1074	0.1289	8.51×10^{-18}	0.1697	0.0308	3.66×10^{-8}	-0.1533	0.0278	3.66×10^{-8} (2.00×10^{-6})
	Albuminuria	-0.0015	0.0001	3.37×10^{-90}	-1.1074	0.1289	8.51×10^{-18}	-0.0158	0.0027	6.09×10^{-9}	0.0143	0.0025	6.09×10^{-9} (1.18×10^{-10})
Hematocrit	eGFR	-0.4312	0.0217	2.88×10^{-87}	-0.1497	0.0208	6.34×10^{-13}	-1.1074	0.1289	8.51×10^{-18}	7.3828	0.8590	8.51×10^{-18} (7.62×10^{-20})
	Uric acid	0.0405	0.0011	1.19×10^{-285}	-0.1492	0.0212	2.08×10^{-12}	0.0704	0.0067	4.02×10^{-26}	-0.4726	0.0447	4.02×10^{-26} (5.12×10^{-31})
	Gout	-0.0108	0.0054	0.0467	-0.1497	0.0208	6.34×10^{-13}	0.1697	0.0308	3.66×10^{-8}	-1.1312	0.2054	3.66×10^{-8} (4.56×10^{-8})
	Albuminuria	0.0003	0.0005	0.5090	-0.1497	0.0208	6.34×10^{-13}	-0.0158	0.0027	6.09×10^{-9}	0.1054	0.0181	6.09×10^{-9} (6.66×10^{-9})
Uric acid	eGFR	-0.0150	0.0002	$< 10^{-307}$	0.0704	0.0067	4.02×10^{-26}	-1.0485	0.1317	1.71×10^{-15}	-14.9790	1.8812	1.71×10^{-15} (8.25×10^{-18})
	Hematocrit	0.0405	0.0011	1.19×10^{-285}	0.0704	0.0067	4.02×10^{-26}	-0.1492	0.0212	2.08×10^{-12}	-2.1316	0.3032	2.08×10^{-12} (2.42×10^{-17})
	Gout	0.6509	0.0146	$< 10^{-307}$	0.0779	0.0068	3.05×10^{-30}	0.1697	0.0308	3.66×10^{-8}	2.1755	0.3951	3.66×10^{-8} (0.0006)
	Albuminuria	0.1308	0.0087	1.07×10^{-50}	0.0704	0.0067	4.02×10^{-26}	-0.0156	0.0027	1.32×10^{-8}	-0.2223	0.0391	1.32×10^{-8} (3.73×10^{-10})
Gout	eGFR	-7.9984	0.4028	1.53×10^{-87}	0.1697	0.0308	3.66×10-^{8}	-1.1074	0.1289	8.51×10^{-18}	-6.5143	0.7580	8.51×10^{-18} (1.76×10^{-16})
	Hematocrit	-0.2942	0.0652	6.00×10^{-6}	0.1697	0.0308	3.66×10^{-8}	-0.1497	0.0208	6.34×10^{-13}	-0.8805	0.1224	6.34×10^{-13} (1.17×10^{-12})
	Uric acid	1.0984	0.0210	$< 10^{-307}$	0.1697	0.0308	3.66×10^{-8}	0.0779	0.0068	3.05×10^{-30}	0.4583	0.0401	3.05×10^{-30} (2.23×10^{-26})
	Albuminuria	0.0725	0.0085	1.67×10^{-17}	0.1697	0.0308	3.66×10^{-8}	-0.0158	0.0027	6.09×10^{-9}	-0.0930	0.0160	6.09×10^{-9} (2.23×10^{-9})
Albuminuria	eGFR	-3.3546	0.1664	3.37×10^{-90}	-0.0158	0.0027	6.09×10^{-9}	-1.1074	0.1289	8.51×10^{-18}	69.2141	8.0533	8.51×10^{-18} (1.74×10^{-19})
	Hematocrit	0.0178	0.0269	0.5090	-0.0158	0.0027	6.09×10^{-9}	-0.1497	0.0208	6.34×10^{-13}	9.3548	1.3003	6.34×10^{-13} (6.92×10^{-13})
	Uric acid	0.1308	0.0087	1.07×10^{-50}	-0.0156	0.0027	1.32×10^{-8}	0.0704	0.0067	4.02×10^{-26}	-4.4014	0.4162	4.02×10^{-26} (1.21×10^{-27})
	Gout	0.1856	0.0361	2.63×10^{-7}	-0.0158	0.0027	6.09×10^{-9}	0.1697	0.0308	3.66×10^{-8}	-10.6054	1.9259	3.66×10^{-8} (1.50×10^{-8})

Abbreviations as in Table 1.

IV_A: Instrumental variables for G_A.

*Adjusted for age, sex, smoking, and body mass index (BMI).

**After further adjustment of T_A

Table S6. *MUC1* DNA methylation sites and the association with functional *MUC1* polymorphisms

IlmnID	MAPINFO	UCSC_RefGene_ Name	UCSC_RefGene_ Group	Relation_to_UCSC_ CpG_Island	Regulatory_Feature_ Group	*MUC1* rs12411216				*MUC1* rs4072037			
						beta	se	*P*	*Adjusted P*	beta	se	*P*	*Adjusted P*
cg13804478	155158319	*MUC1*	3'UTR			-0.0140	0.0051	0.0060	0.1380	-0.0142	0.0051	0.0053	0.1219
cg06339768	155159820	*MUC1*	Body	N-shelf		-0.0675	0.0058	1.33×10^{-30}	3.06×10^{-29}	-0.0669	0.0058	4.41×10^{-30}	1.01×10^{-28}
cg19011149	155160273	*MUC1*	Body	N-shelf		-0.0985	0.0055	8.77×10^{-66}	2.02×10^{-64}	-0.0996	0.0055	1.84×10^{-67}	4.23×10^{-66}
cg15646096	155160365	*MUC1*	Body	N-shelf		-0.1153	0.0045	2.83×10^{-120}	6.51×10^{-119}	-0.1162	0.0045	5.83×10^{-123}	1.34×10^{-121}
cg00126087	155160941	*MUC1*	Body	N-shelf		-0.0840	0.0076	1.39×10^{-27}	3.20×10^{-26}	-0.0851	0.0076	2.54×10^{-28}	5.84×10^{-27}
cg06216400	155161161	*MUC1*	Body	N-shelf		0.0168	0.0097	0.0827	0.9999	0.0165	0.0097	0.0881	0.9999
cg15699386	155161211	*MUC1*	Body	N-shelf		0.0525	0.0115	6.00×10^{-6}	1.38×10^{-4}	0.0549	0.0115	2.00×10^{-6}	4.60×10^{-5}
cg00930306	155161221	*MUC1*	Body	N-shelf		0.0081	0.0118	0.4919	0.9999	0.0081	0.0118	0.4882	0.9999
cg20949223	155161679	*MUC1*	Body	N-shore		0.0244	0.0114	0.0317	0.7291	0.0204	0.0114	0.0732	0.9999
cg24512973	155161784	*MUC1*	Body	N-shore		0.0210	0.0078	0.0071	0.1633	0.0185	0.0078	0.0176	0.4048
cg18804777	155161833	*MUC1*	Body	N-shore		0.0218	0.0075	0.0039	0.0897	0.0208	0.0075	0.0059	0.1357
cg22500132	155162752	*MUC1*	TSS200	N-shore	Promoter-associated	-0.0453	0.0122	0.0002	0.0046	-0.0431	0.0122	0.0004	0.0092
cg22531371	155162756	*MUC1*	TSS200	N-shore	Promoter-associated	-0.0276	0.0082	0.0008	0.0184	-0.0272	0.0082	0.0009	0.0207
cg02386822	155162871	*MUC1*	TSS200	N-shore	Promoter-associated	0.0005	0.0104	0.9637	0.9999	0.0006	0.0104	0.9570	0.9999
cg11011735	155162891	*MUC1*	TSS200	N-shore	Promoter-associated	-0.0045	0.0067	0.5060	0.9999	-0.0042	0.0067	0.5343	0.9999
cg07399355	155163013	*MUC1*	TSS1500	N-shore	Promoter-associated	-0.0136	0.0122	0.2655	0.9999	-0.0165	0.0122	0.1767	0.9999
cg23256951	155163205	*MUC1*	TSS1500	N-shore	Promoter-associated	-0.0366	0.0272	0.1790	0.9999	-0.0381	0.0272	0.1610	0.9999
cg19755544	155163240	*MUC1*	TSS1500	N-shore	Promoter-associated	0.0161	0.0203	0.4263	0.9999	0.0219	0.0202	0.2786	0.9999
cg17257175	155163542	*MUC1;MIR92B*	TSS1500	Island	Promoter-associated	0.0123	0.0152	0.4190	0.9999	0.0127	0.0152	0.4032	0.9999
cg06420088	155163590	*MUC1;MIR92B*	TSS1500	Island	Promoter-associated	0.0032	0.0071	0.6479	0.9999	0.0016	0.0071	0.8221	0.9999
cg03055449	155163661	*MUC1;MIR92B*	TSS1500	Island	Promoter-associated	-0.0269	0.0206	0.1917	0.9999	-0.0266	0.0206	0.1959	0.9999
cg27538026	155164351	*MIR92B*	TSS1500	Island	Promoter-associated	-0.0001	0.0088	0.9952	0.9999	-0.001	0.0088	0.9097	0.9999
cg15434337	155164363	*MIR92B*	TSS1500	Island	Promoter-associated	-0.0030	0.0075	0.6914	0.9999	-0.0043	0.0075	0.5695	0.9999

The UCSC_RefGene_Name, UCSC_RefGene_Group, Relation_to_UCSC_CpG_Island, and Regulatory_Feature_Group are derived from the UCSC (University of California Santa Cruz) database. https://genome.ucsc.edu/

Adjusted P value: with Bonferroni correction, n = 23

Table S7. Association between *MUC1* methylation site cg15646096 and clinical phenotypes and laboratory parameters (N = 1,686)

Clinical and laboratory parameters		Adjusted for sex age and BMI SMK			
		beta	SE	*P* value	*Adjusted P*
Anthropology	Age (years)	-27.3823	1.9005	1.83×10^{-44}	4.58×10^{-43}
	Body mass index (kg/m2)	0.7803	0.6729	0.2464	0.9999
Blood Pressure	Systolic BP* (mmHg)	-0.2767	2.858	0.9229	0.9999
	Diastolic BP* (mmHg)	1.4842	1.9683	0.4509	0.9999
	Mean BP* (mmHg)	0.8972	2.0985	0.6690	0.9999
Lipid profiles	Total cholesterol**** (mg/dL)	0.0055	0.0156	0.7271	0.9999
	HDL-cholesterol**** (mg/dL)	0.0049	0.0185	0.7915	0.9999
	LDL-cholesterol**** (mg/dL)	0.0068	0.0242	0.7792	0.9999
	Triglyceride**** (mg/dL)	-0.0413	0.0438	0.3454	0.9999
Glucose metabolism	Fasting plasma glucose** (mg/dL)	4.4858	3.0116	0.1365	0.9999
	HbA1C** (%)	0.1478	0.1155	0.2008	0.9999
Renal function	Creatinine (mg/dL)	-0.027	0.0265	0.3082	0.9999
	eGFR (mL/min/1.73 m^2)	5.552	2.9126	0.0568	0.9999
	BUN (mg/dL)	-0.3075	0.6445	0.6333	0.9999
	Albuminuria (mg/L)	31.2145	20.4355	0.1268	0.9999
Liver function	AST (U/L)	-3.1678	1.8285	0.0834	0.9999
	ALT (U/L)	-7.314	3.5131	0.0375	0.9375
	γGT (U/L)	-9.4623	7.2968	0.1949	0.9999
	Serum albumin (g/dL)	-0.056	0.0474	0.2375	0.9999
	Total bilirubin (mg/dL)	-0.0242	0.0529	0.6476	0.9999
Hematological parameters	Leukocyte count (10^3/μL)	0.7979	0.2811	0.0046	0.1150
	Hematocrit (%)	-0.8158	0.6827	0.2323	0.9999
	Platelet count (10^3/μL)	18.0503	9.9798	0.0707	0.9999
	Red blood cell count (10^6/μL)	-0.0204	0.0805	0.7998	0.9999
	Hemoglobin (g/dL)	-0.0798	0.2265	0.7247	0.9999

Abbreviations as in Table 1.
P: adjusted for age, BMI and current smoking
Age: adjusted for BMI, sex and current smoking.
BMI: adjusted for age, sex and smoking
* were analyzed with the exclusion of participants with previous history of hypertension;
** were analyzed with the exclusion of participants with previous history of diabetes mellitus;
*** were analyzed with the exclusion of participants with previous history of gout
**** were analyzed with the exclusion of participants with previous history of hyperlipidemia
Data are presented as mean ± SD, percentage, or median (interquartile range) as appropriate
Adjusted P value: with Bonferroni correction, n = 25

Table S8. Association between *MUC1* methylation site cg15646096 and Atherosclerotic risk factors (N = 1686)

	beta	SE	*P* value	*Adjusted P*
Diabetes mellitus (%)	-0.1973	0.8223	0.8103	0.9999
Hypertension (%)	0.3247	0.5294	0.5397	0.9999
Gout (%)	0.7026	1.1057	0.5252	0.9999
Current drinking (%)	-0.1973	0.8223	0.8103	0.9999
Current smoking (%)	-1.1236	0.5157	0.0293	0.1465

P value: Adjusted for age , sex, body mass index and current smoking
Adjusted P value: with Bonferroni correction, n = 5

Table S9. *MUC1* functional variant table

Chr	Gene	rs number	Position CHR.37	Location relative to gene	Genotype (Ref/Alt)	Function	SNPinfo	Regulomn DB rank	Regulomn DB score	MAF
1	*MUC1*	rs4072037	155162067	3767	A/G	Synonymous variant	Splicing (ESE or ESS)	1f	0.55436	C : 0.2025
1	*MUC1*	rs12411216	155164480	6180	C/A	3' untranslated region	TFBS	--	--	A : 0.2052

Chr: chromosome, Ref/Alt: reference allele/alternative allele, ESE: exon splicing enhancer, ESS: exon splicing silencer, TFBS: transcription factor binding site, MAF: minor allele frequency

Figure S1. Regional-wide association studies on chromosome 1q22 for waist circumference without (A) or with (B) conditional analysis.

(A)

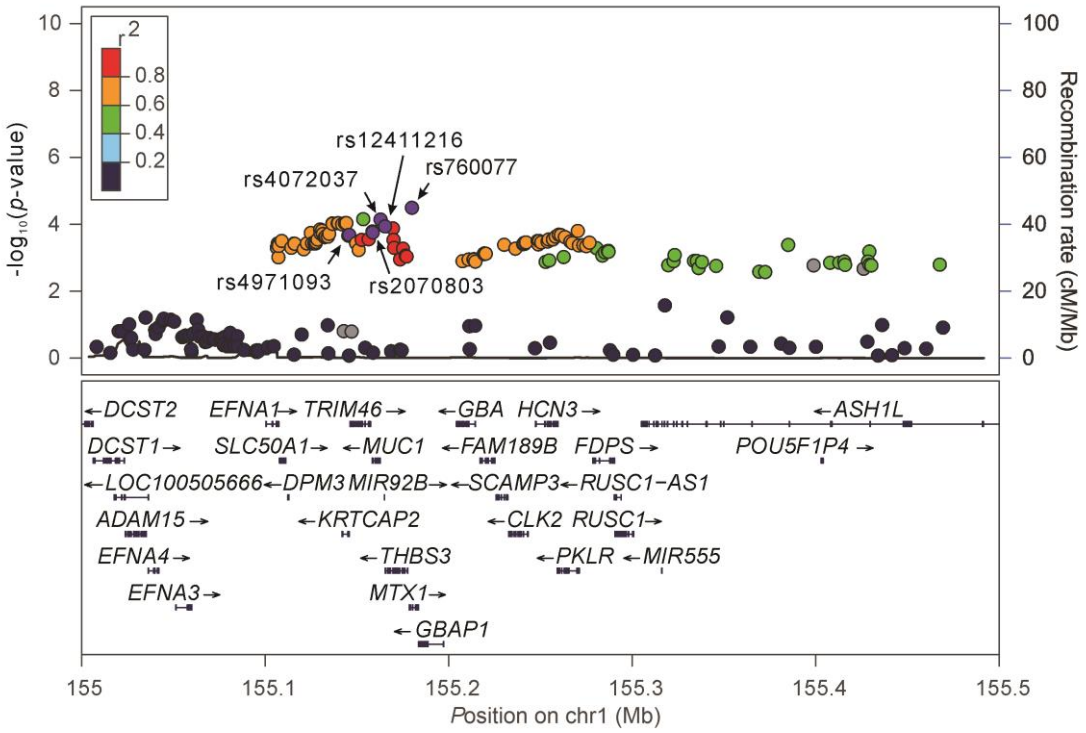

(B)

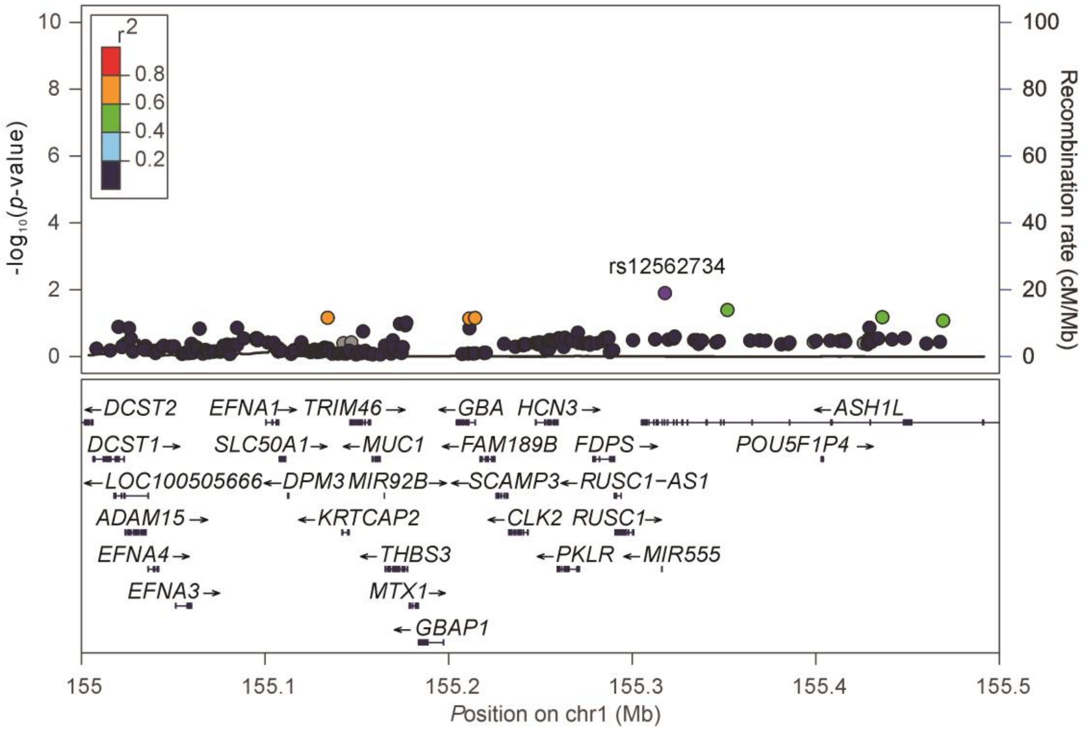

Figure S2. Regional-wide association studies on chromosome 1q22 for hemoglobin A1C without (A) or with (B) conditional analysis.

(A)

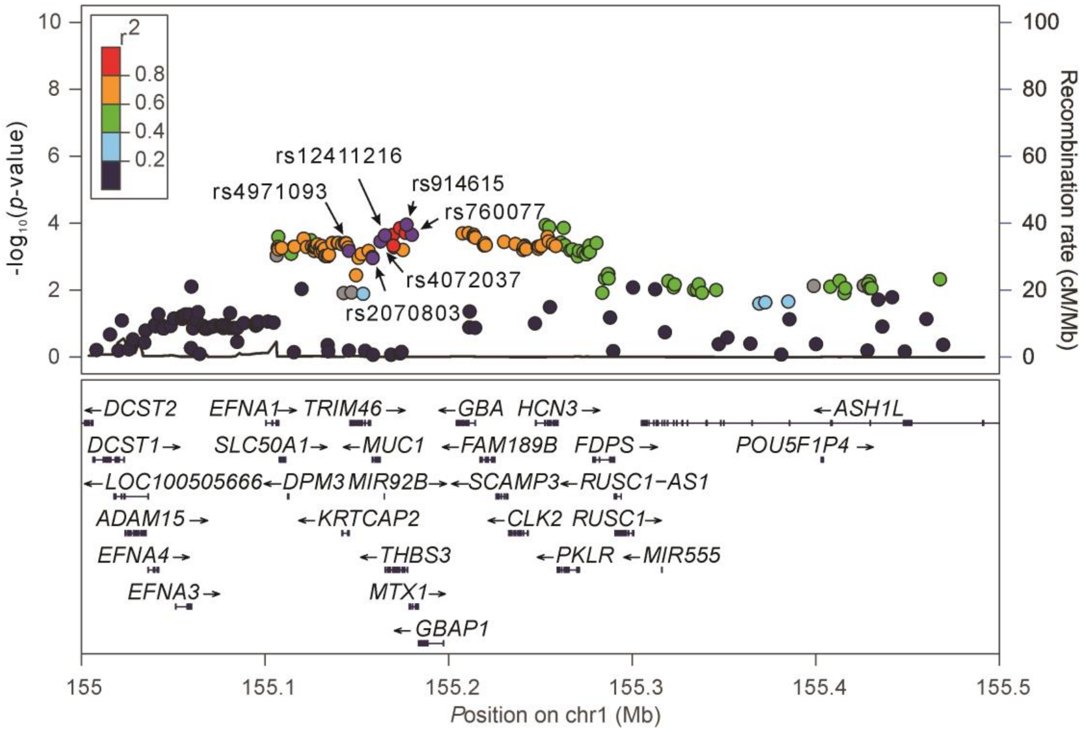

(B)

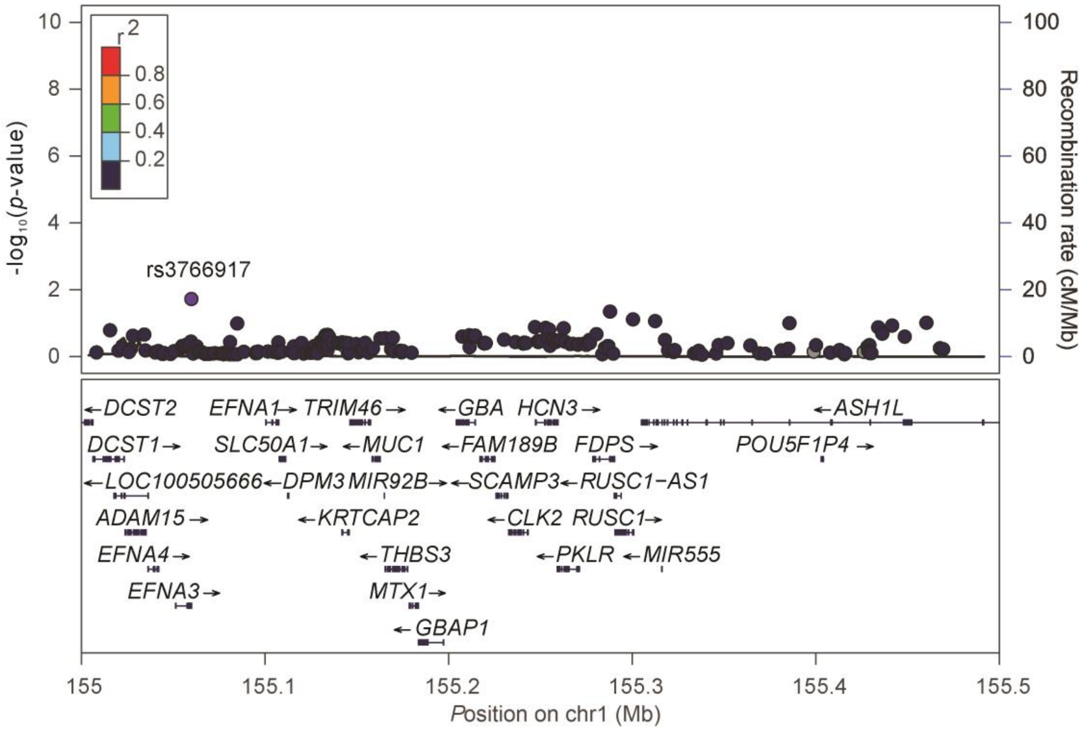

Figure S3. Regional-wide association studies on chromosome 1q22 for serum uric acid levels without (A) or with (B) conditional analysis.

(A)

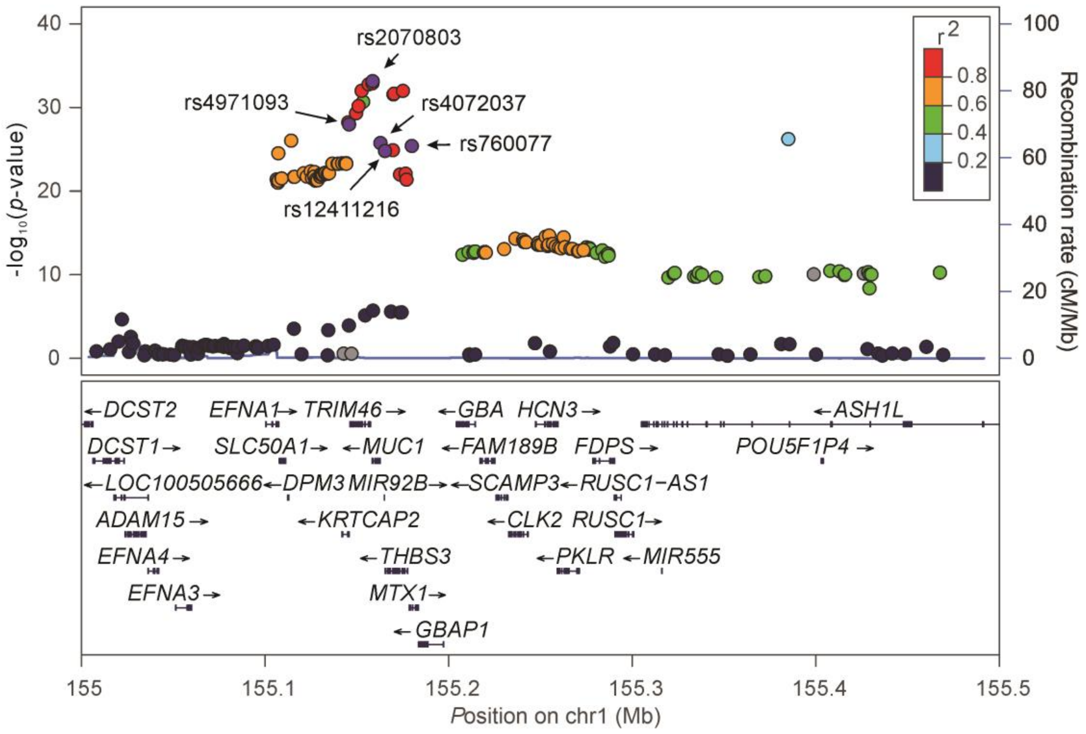

(B)

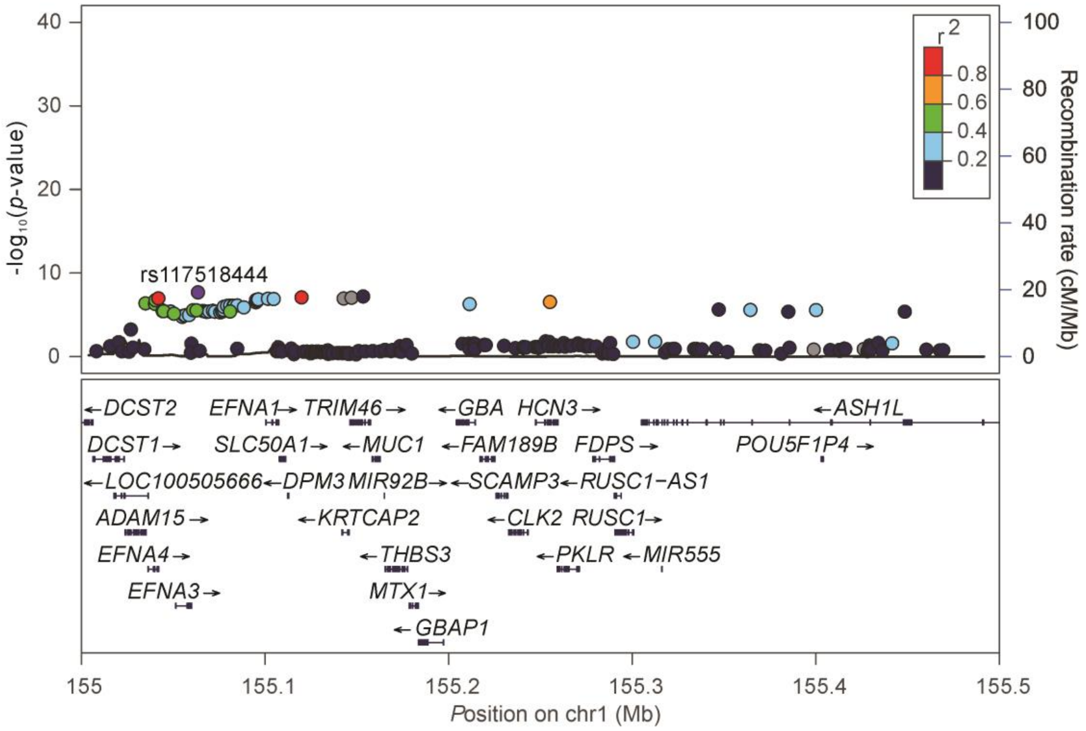

Figure S4. Regional-wide association studies on chromosome 1q22 for serum creatinine level.

(A)

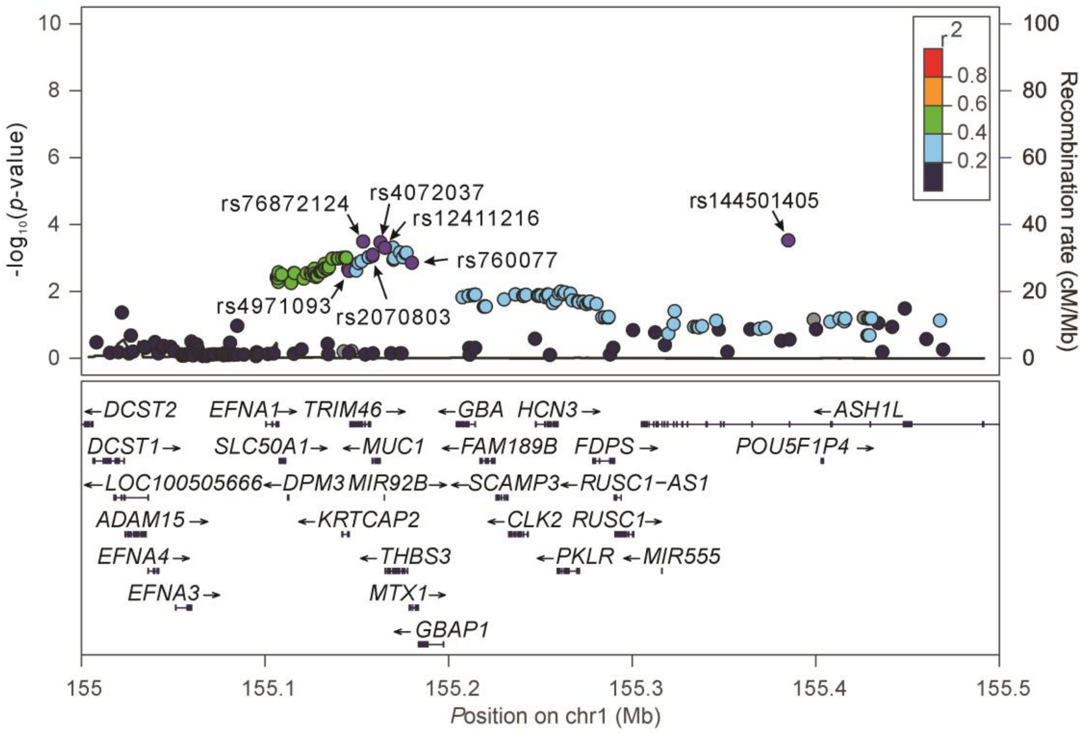

(B)

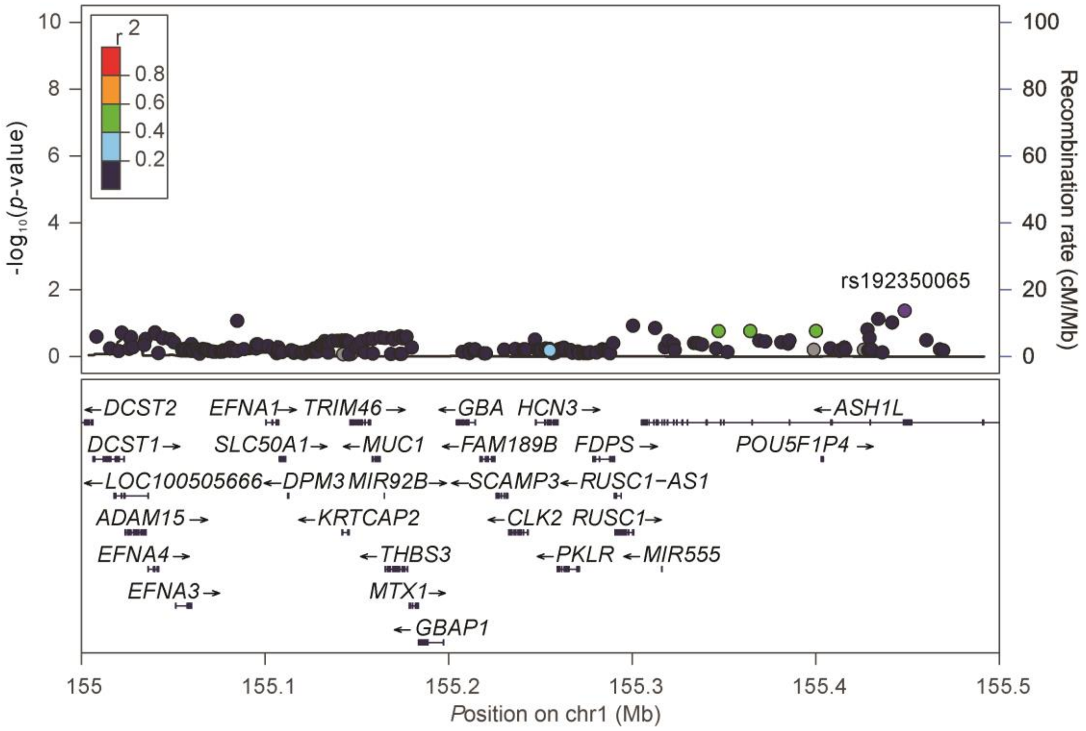

Figure S5. Regional-wide association studies on chromosome 1q22 for estimated glomerular filtration rate without (A) or with (B) conditional analysis.

(A)

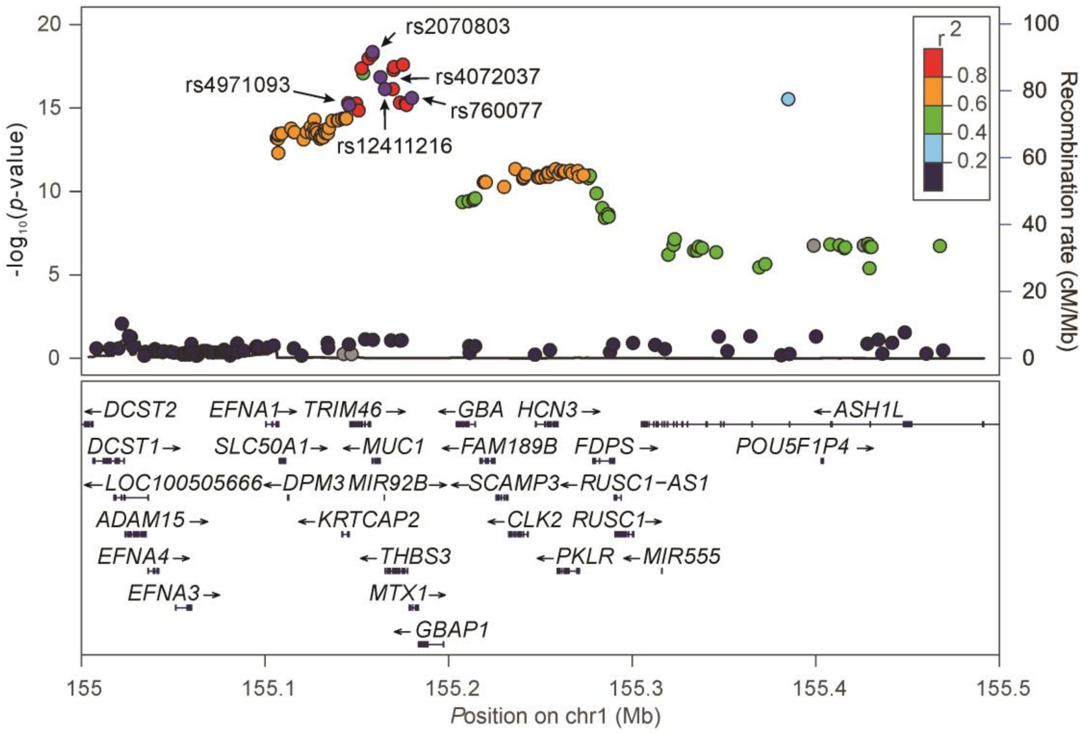

(B)

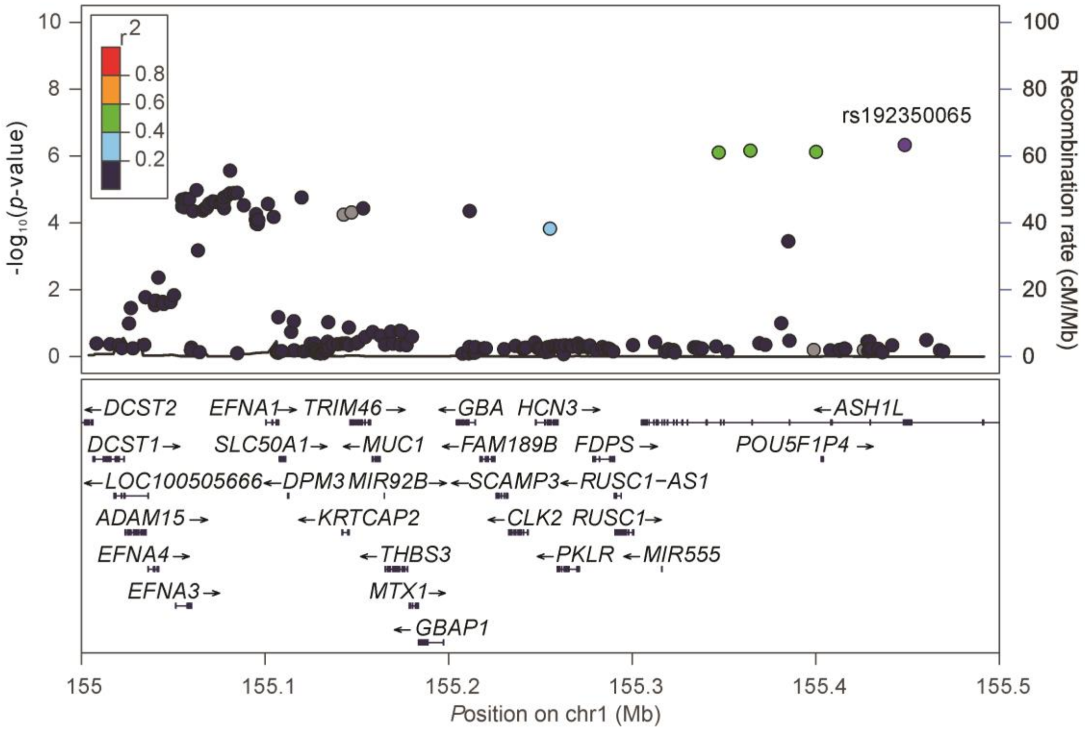

Figure S6. Regional-wide association studies on chromosome 1q22 for blood urea nitrogen without (A) or with (B) conditional analysis.

(A)

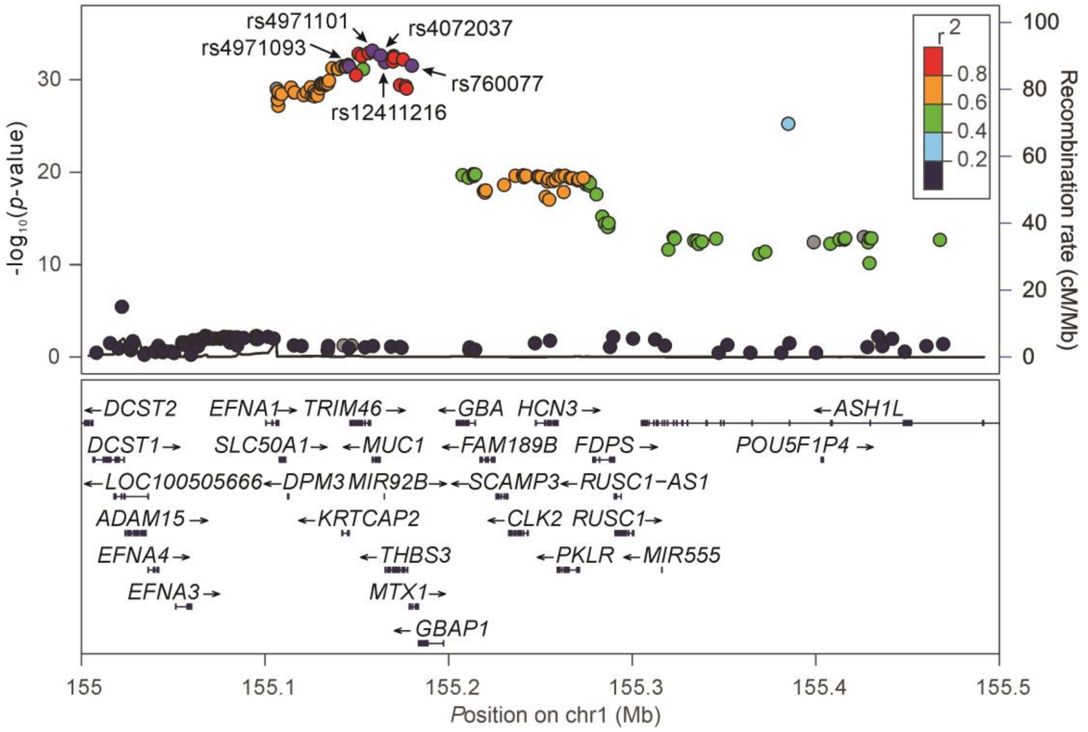

(B)

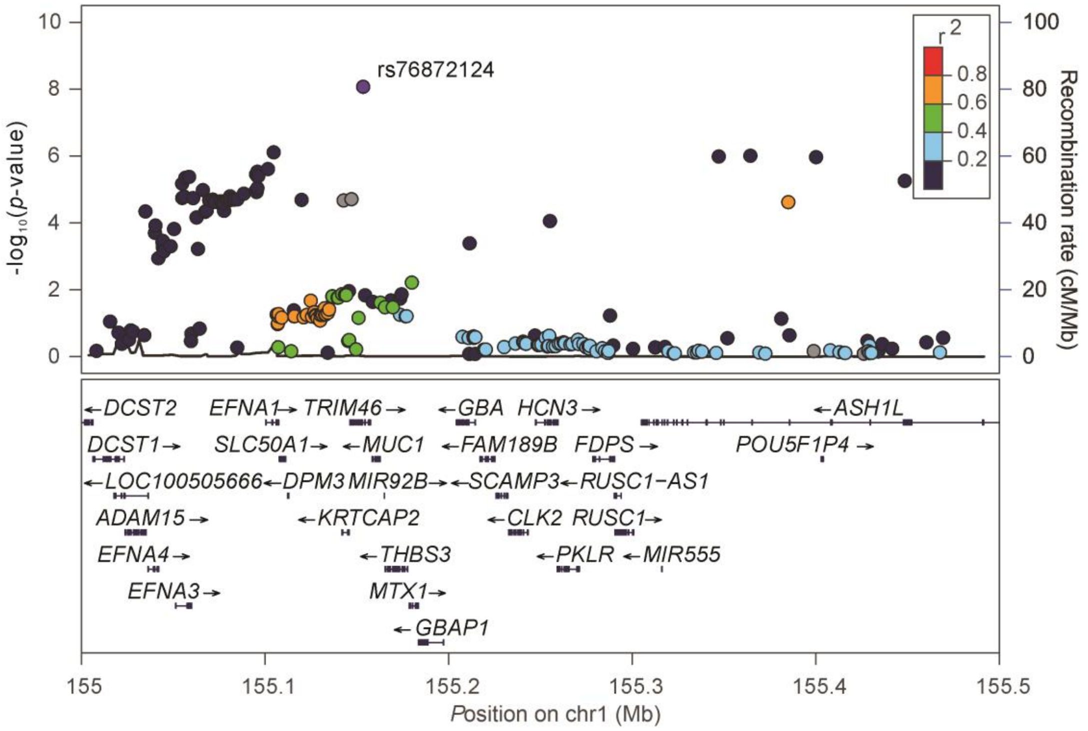

Figure S7. Regional-wide association studies on chromosome 1q22 for albuminuria without (A) or with (B) conditional analysis.

(A)

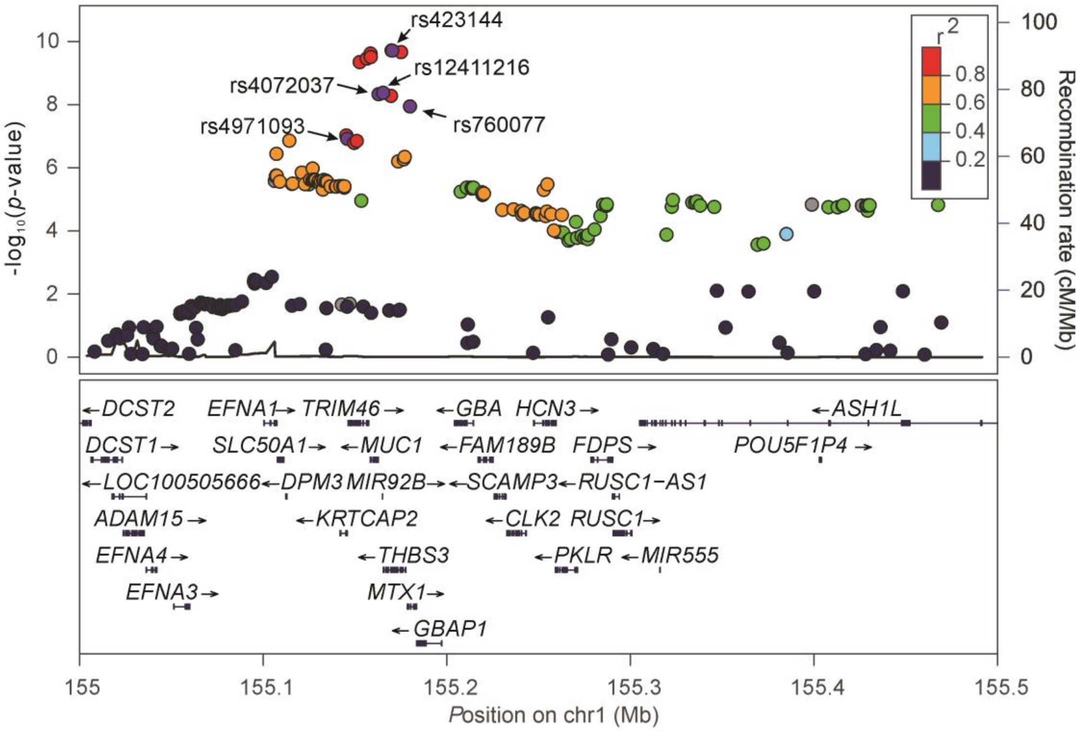

(B)

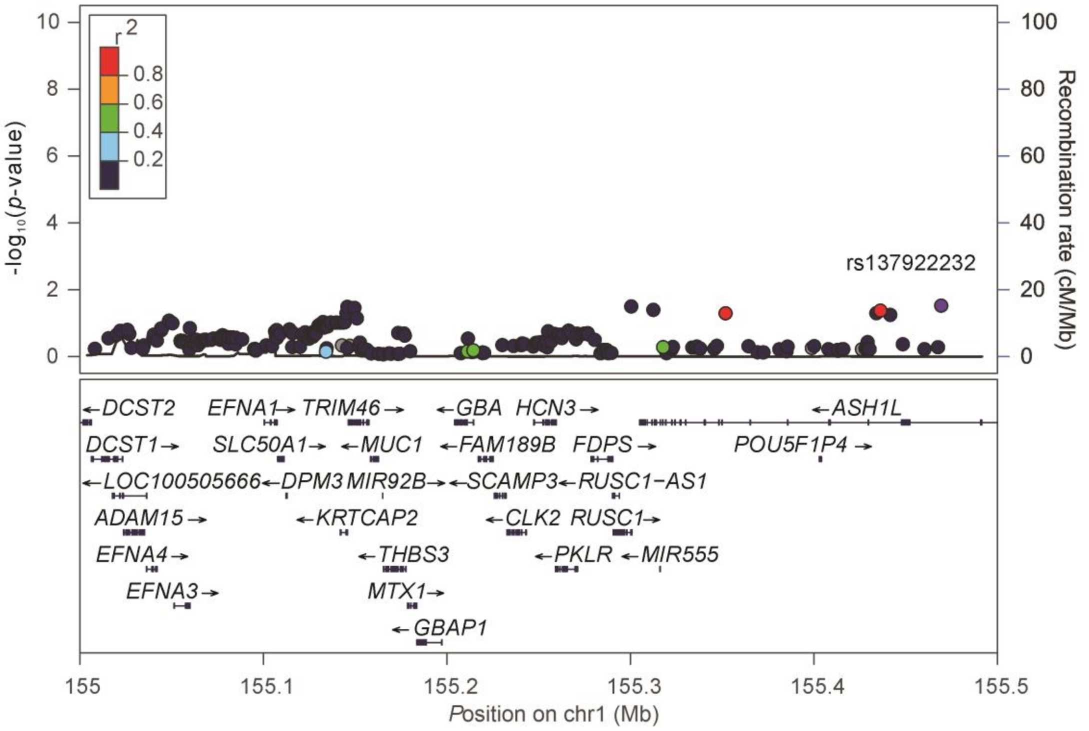

Figure S8. Regional-wide association studies on chromosome 1q22 for hematocrit without (A) or with (B) conditional analysis.

(A)

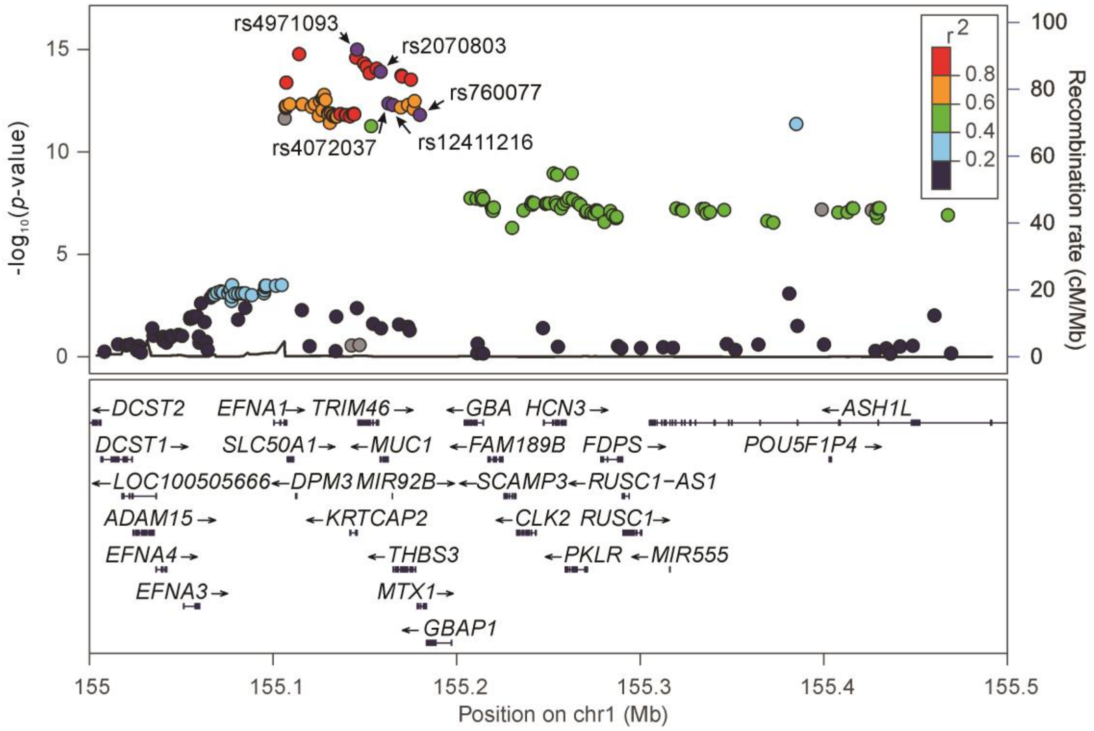

(B)

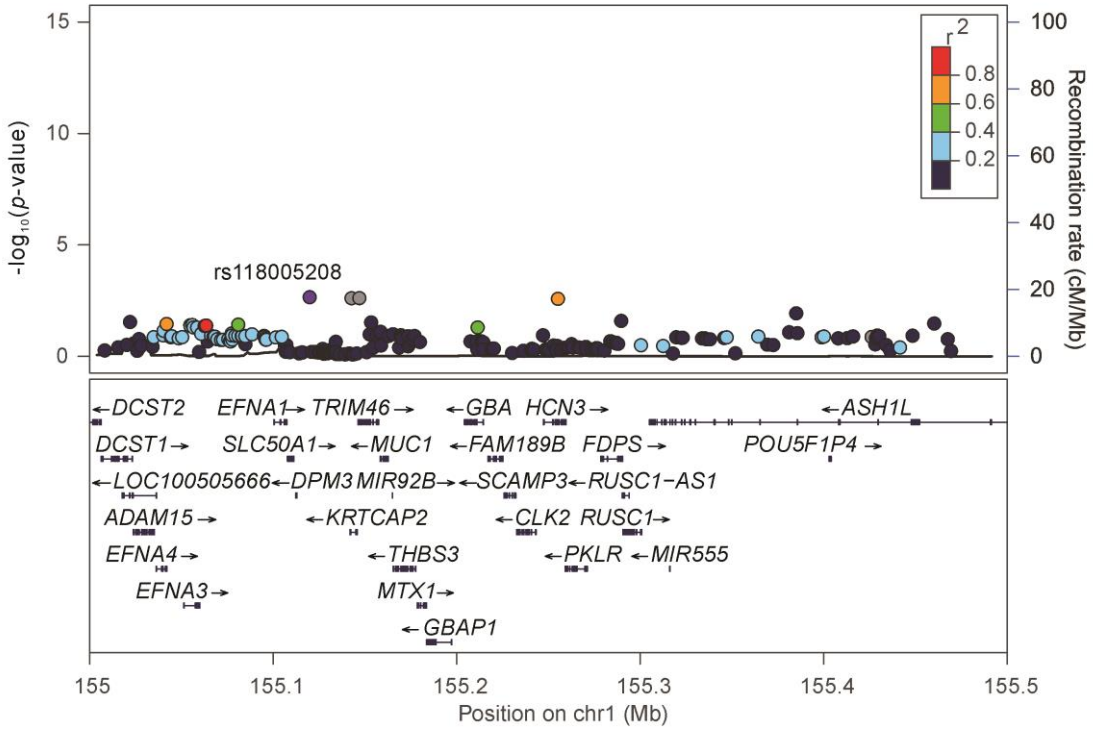

Figure S9. Regional-wide association studies on chromosome 1q22 for red blood cell count without (A) or with (B) conditional analysis.

(A)

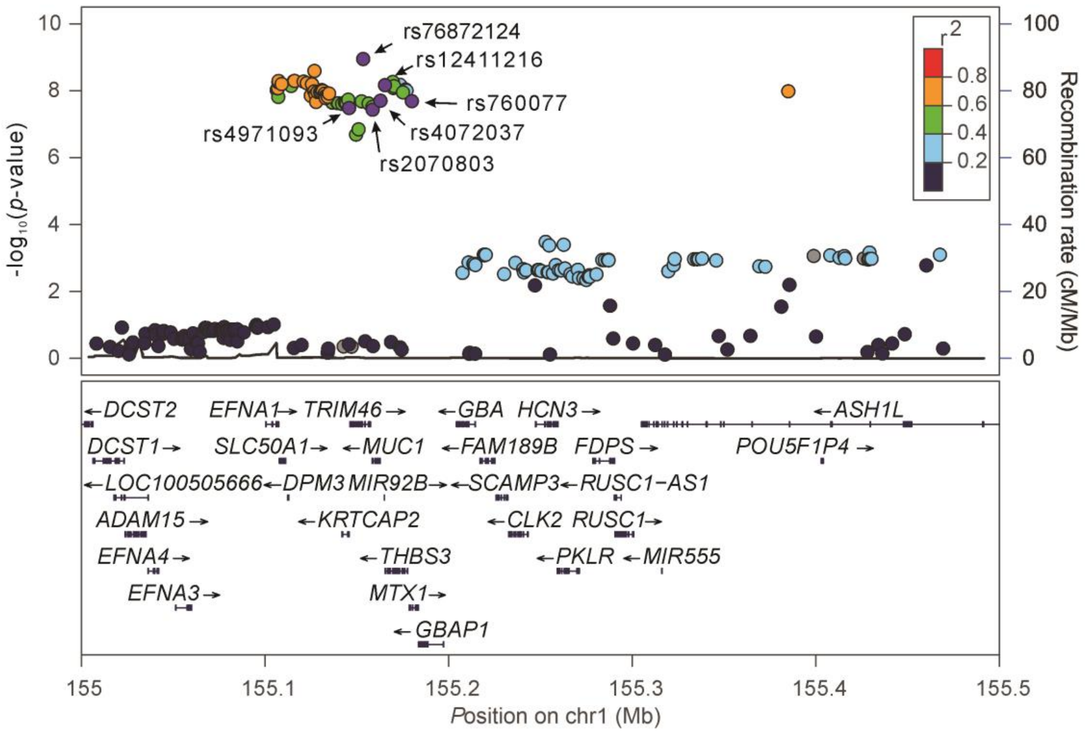

(B)

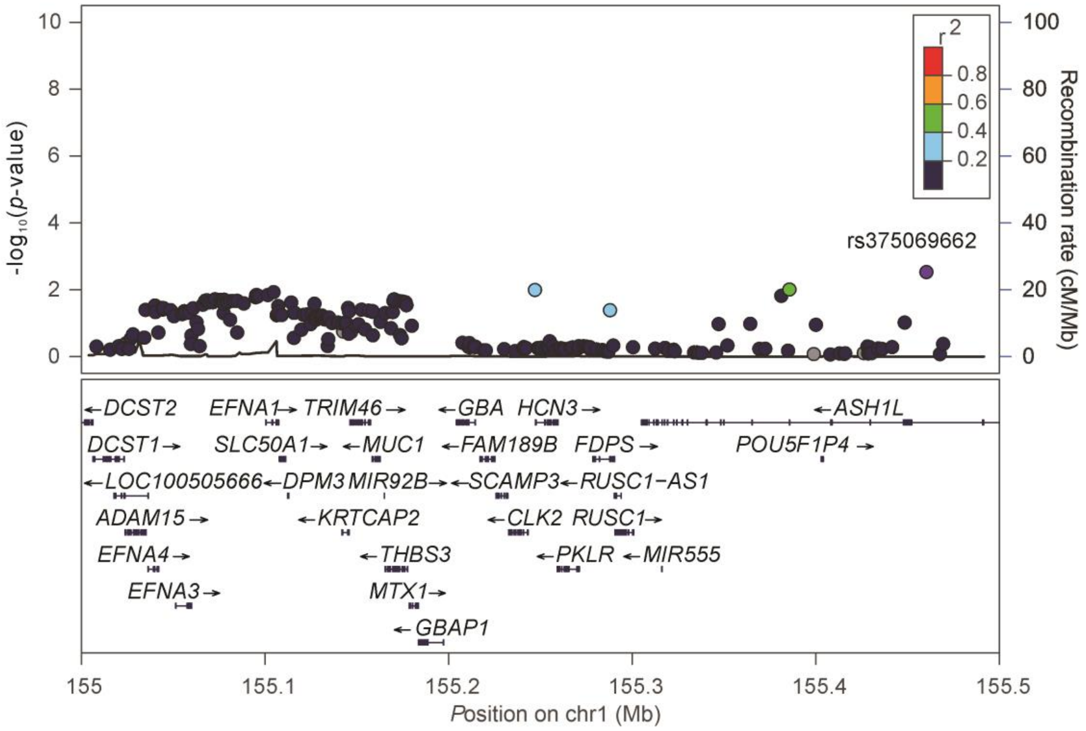

Figure S10. Regional-wide association studies on chromosome 1q22 for hemoglobin without (A) or with (B) conditional analysis.

(A)

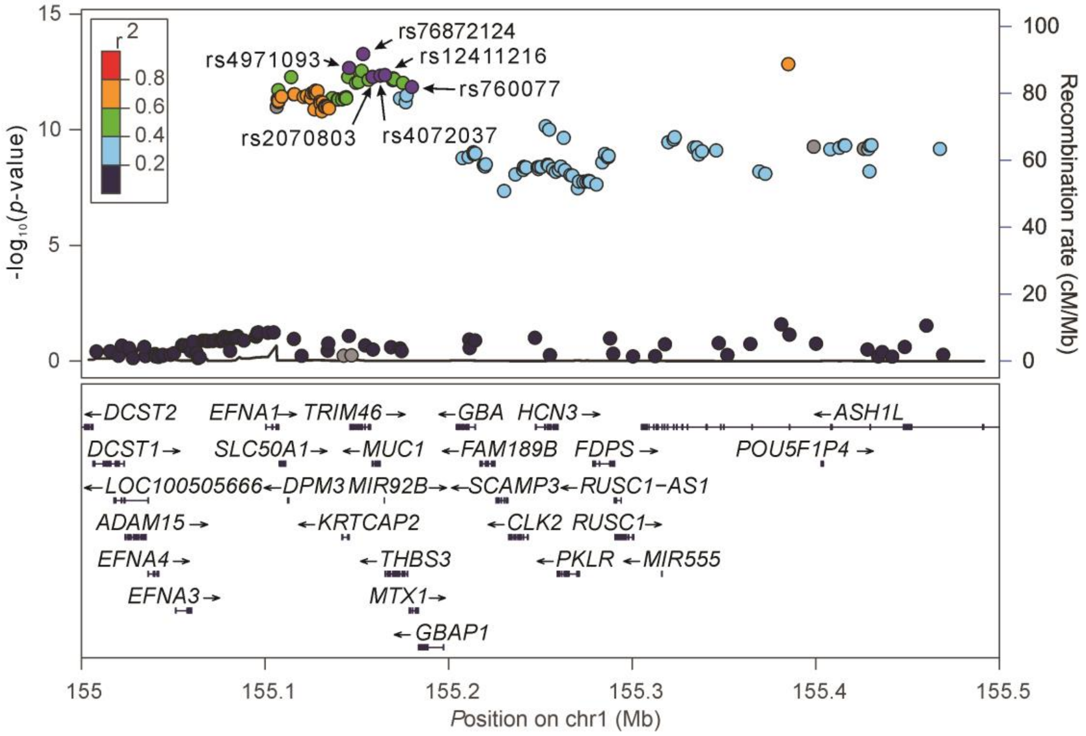

(B)

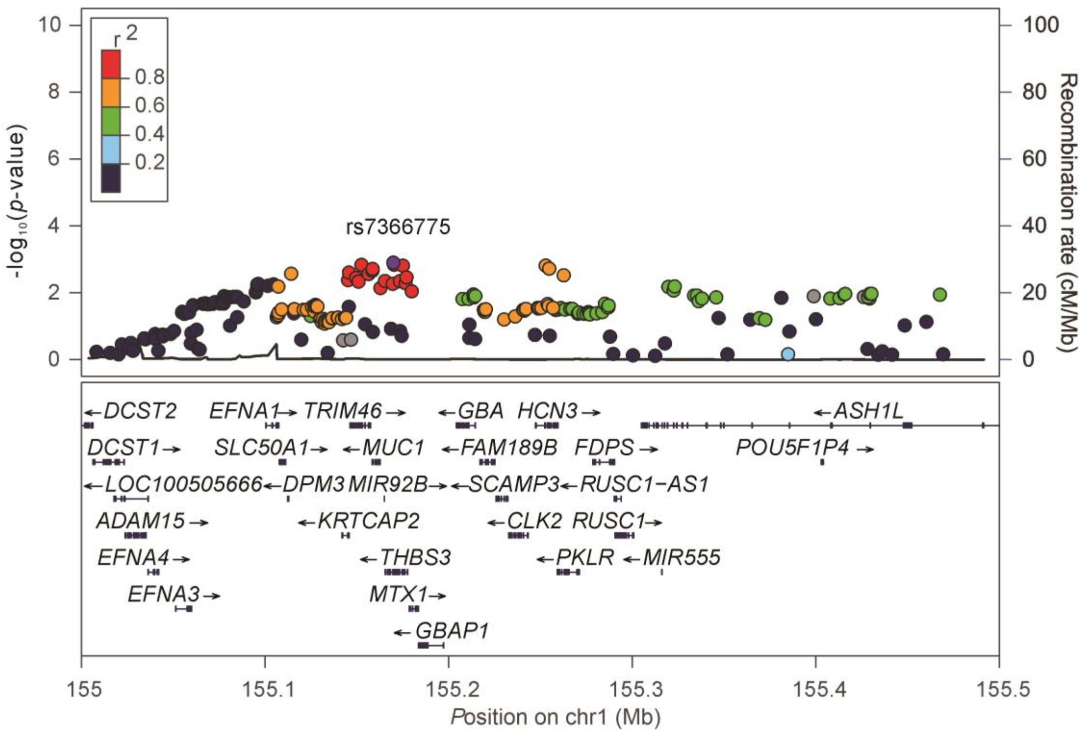

Figure S11. Regional-wide association studies on chromosome 1q22 for gout without (A) or with (B) conditional analysis.

(A)

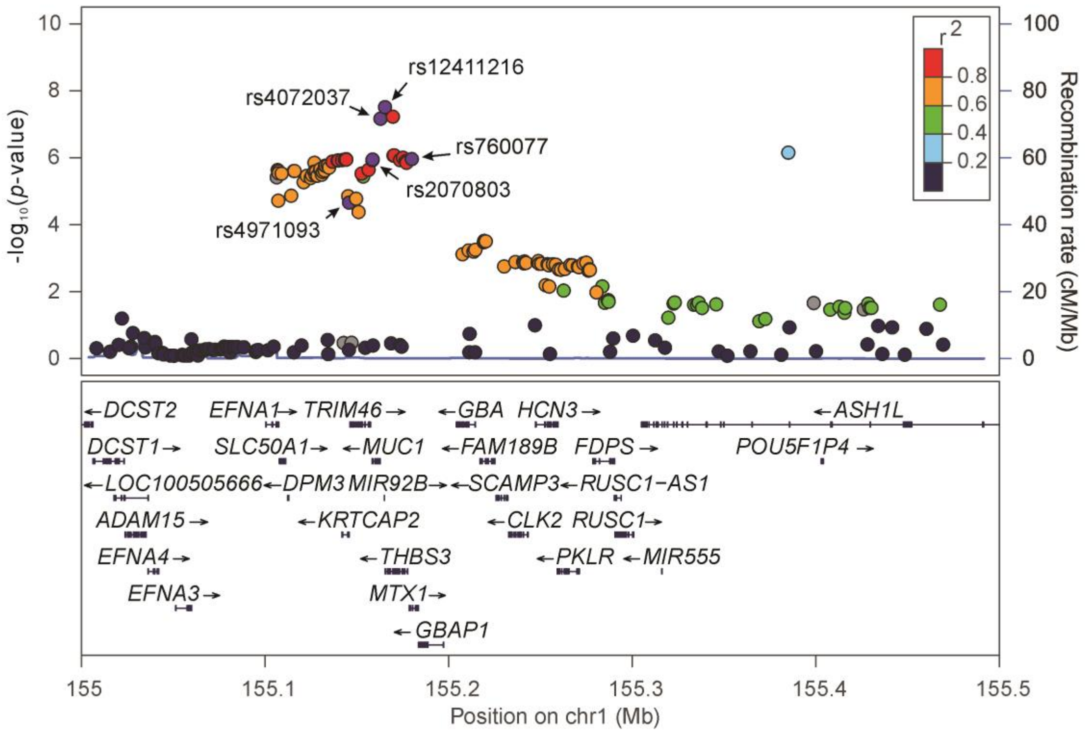

(B)

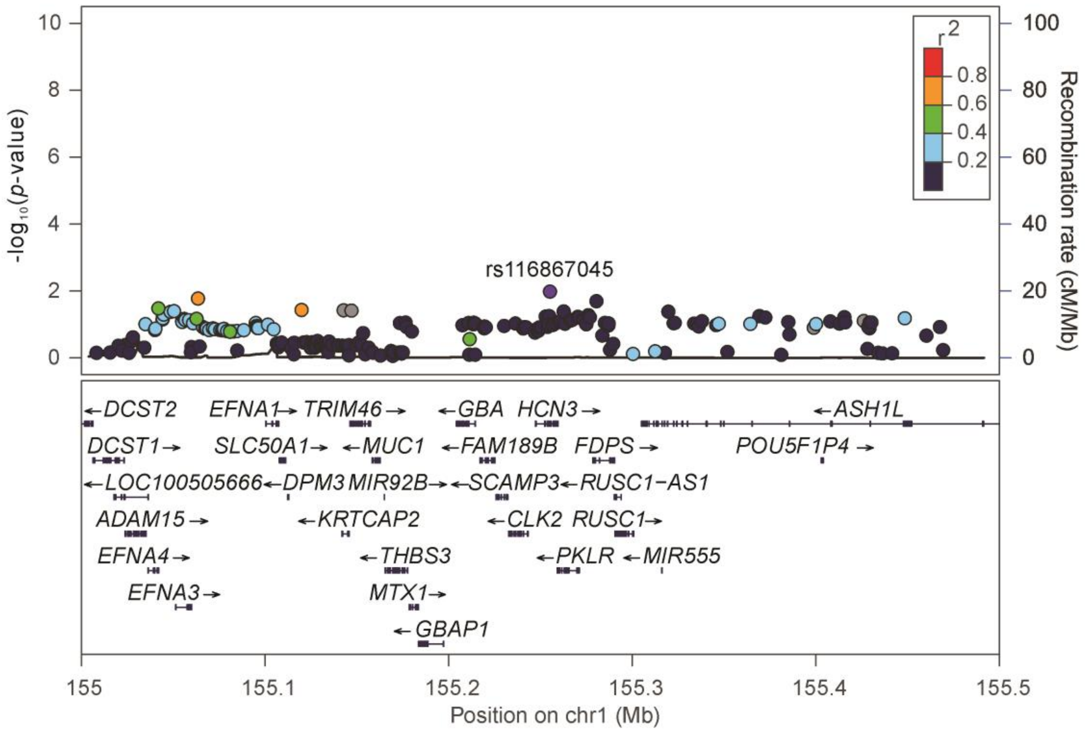

Figure S12. Regional-wide association studies on chromosome 1q22 for microalbuminuria without (A) or with (B) conditional analysis.

(A)

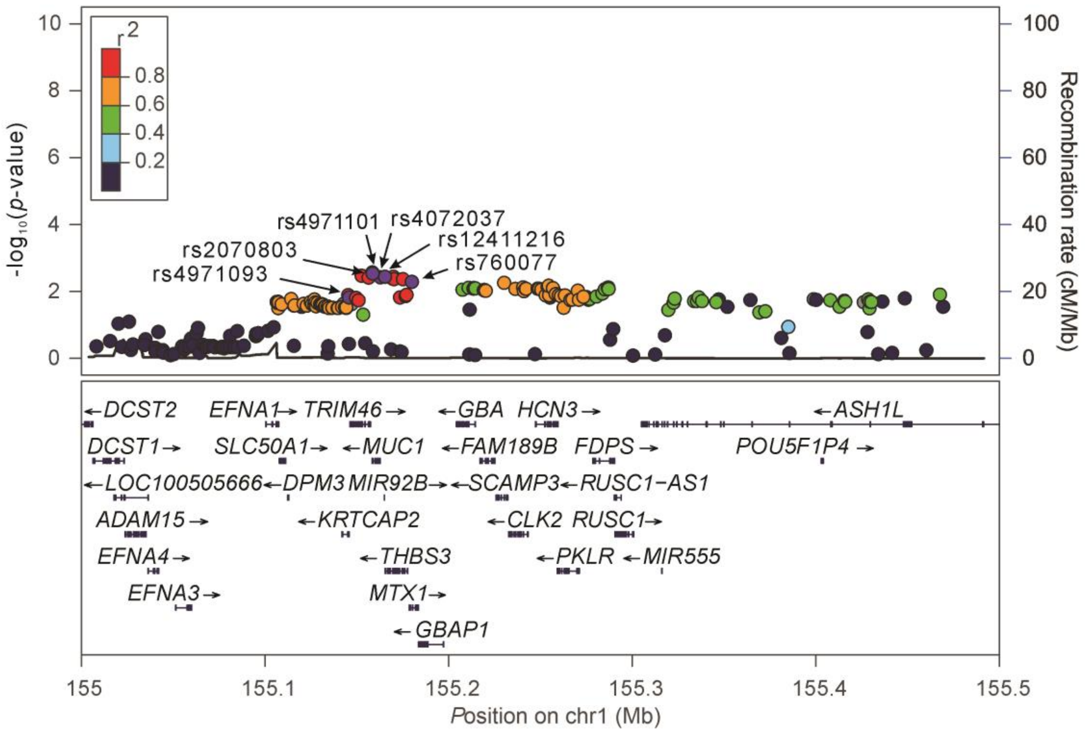

(B)

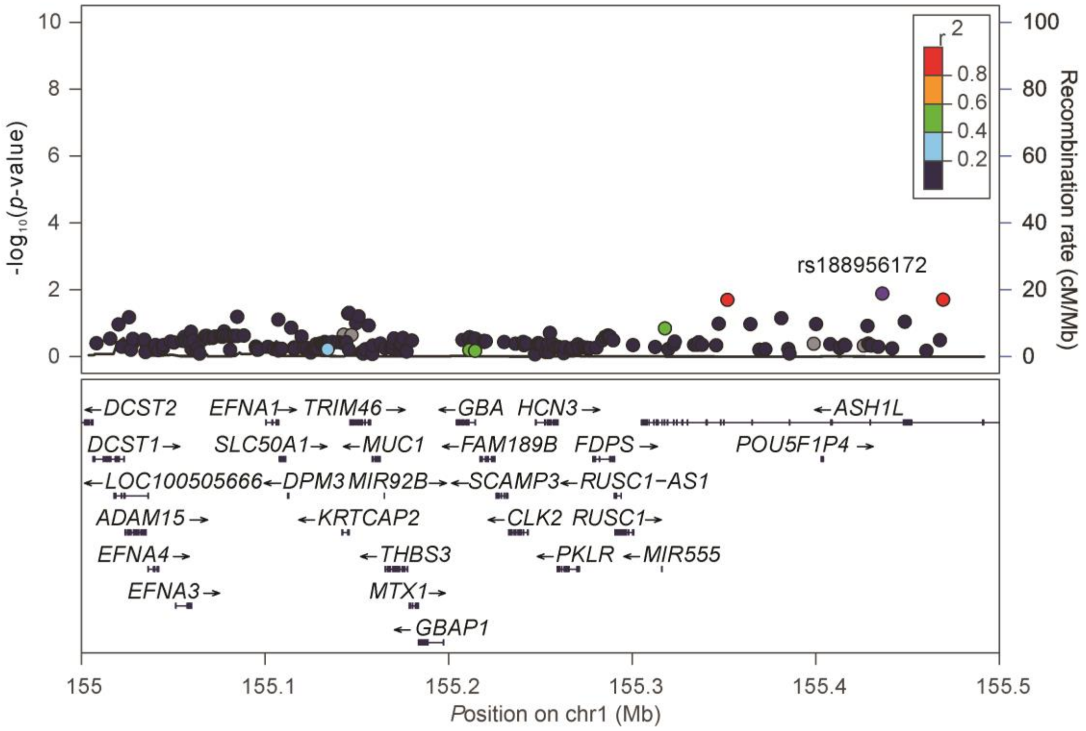

Supplement: Supplementary file 1 [file ijms-22-10641-s001.zip › ijms-1361215-supplementary.pdf]
